# Correlation study on adiponectin gene SNP45 and long-term oxidative stress in patients with diabetes and carotid atherosclerosis

**DOI:** 10.3892/etm.2014.1808

**Published:** 2014-06-25

**Authors:** LIANSHAN PIAO, YANHUA HAN, DAN LI

**Affiliations:** Department of Endocrinology, The Affiliated Hospital of Yanbian University, Yanji, Jilin 133000, P.R. China

**Keywords:** type II diabetes, carotid intima-media thickness, adiponectin single nucleotide polymorphism 45 T/G, long-term oxidative stress

## Abstract

The aim of the present study was to investigate the correlation between the adiponectin gene single nucleotide polymorphism (SNP)45 T/G and long-term oxidative stress in type II diabetes mellitus (T2DM) patients with carotid atherosclerosis. Patients with T2DM were divided into non-carotid atherosclerosis and carotid atherosclerosis groups, which were then subsequently divided into TT and TG + GG groups according to the adiponectin SNP45 T/G genotypes. Enzyme-linked immunosorbent assay, *Taq*Man probe quantitative polymerase chain reaction (PCR), PCR-*Taq*Man, color Doppler and other methods were used to determine the adiponectin levels, gene polymorphisms, acquired mitochondrial DNA (mtDNA) A3243G somatic cell mutation rates and the carotid intima-media thickness. The somatic cell mutation rate of acquired mtDNA A3243A/G in the T2DM carotid atherosclerosis group was significantly higher compared with the group without carotid atherosclerosis. In addition, the acquired mtDNA A3243A/G somatic cell mutation rate in the T2DM carotid atherosclerosis group with the adiponectin gene SNP45 TT genotype was significantly lower compared with the SNP45 TG/GG genotype group. T2DM combined with carotid atherosclerosis was associated with long-term oxidative stress. In addition, adiponectin gene SNP45 T/G was associated with increased mtDNA A3243A/G somatic mutation rates in T2DM patients with carotid atherosclerosis. Therefore, adiponectin gene polymorphisms may lead to diabetes atherosclerosis through oxidative stress.

## Introduction

The morbidity of type 2 diabetes patients developing cardio vascular disease is 2–4 times as compared to people without diabetes. Carotid intima-media thickness (IMT) is a predictor of coronary heart disease and cerebrovascular disease and is used as an early clinical quantitative index for atherosclerosis in diabetic patients. The pathogenesis of atherosclerosis in patients with type II diabetes mellitus (T2DM) is not fully understood. To date, out of the T2DM patients with carotid artery IMT thickening, 35% of them are due to causes of atherosclerosis that are already known and 65% of them are due to gene polymorphisms, oxidative stress and some other factors.

A hyperglycemic state in diabetic patients causes the occurrence and development of atherosclerosis through oxidative stress. Oxidative stress hyperfunction in diabetic patients is four times higher than in individuals without the disease. The current indicators used to determine the level of oxidative stress only reflect the short-term levels. However, the somatic mutation rate of acquired mitochondrial DNA (mtDNA) A3243G may be determined by *Taq*Man probe quantitative polymerase chain reaction (PCR) and this reflects the long-term levels of oxidative stress in patients with T2DM (1,2).

Adiponectin is specifically secreted by fat tissue, thus, systemic oxidative stress primarily originates from adipose tissues. Previous studies (3–7) have demonstrated that adiponectin is associated with oxidative stress, however, the specific mechanism remains unclear. Oxidative stress inhibits adiponectin expression and reduces the production of adiponectin, while adiponectin also inhibits oxidative stress. However, identifying whether adiponectin or oxidative stress is the cause or result has yet to be determined. With regard to the risk factors for coronary artery disease, plasma adiponectin concentrations were found to positively correlate with prostaglandin oxide, a short-term indicator of oxidative stress (7). In addition, decreased levels of adiponectin and secretion imbalance have been shown to be associated with T2DM and atherosclerosis (8–10). Adiponectin may affect the development of chronic complications, including T2DM and macrovascular complications, by means of oxidative stress (7). Previous studies found that in patients with T2DM, single nucleotide polymorphisms (SNPs) of the adiponectin gene at +45 and +276 were associated with risks of coronary heart disease (11–15). In addition, the association between adiponectin levels, gene polymorphisms and T2DM with atherosclerosis have been shown to differ between ethnicities (10,14–16).

In summary, adiponectin is associated with short-term oxidative stress and atherosclerosis. However, to the best of our knowledge, no studies have investigated the correlations between adiponectin levels, gene polymorphisms, long-term oxidative stress and atherosclerosis. Adiponectin gene polymorphisms have been shown to differ between ethnicities and interactions may exist in ethnic genetic heterogeneity or genetic and environmental factors or intergenetic interactions. Therefore, in the present study, enzyme-linked immunosorbent assay, *Taq*Man probe quantitative PCR, PCR-*Taq*Man technology, color Doppler and other methods were used to analyze the correlations among T2DM, adiponectin gene polymorphisms, acquired somatic mutation rates of mtDNA A3243G and carotid IMT in Korean and Han populations in the Yanbian region. Thus, the present study aimed to determine whether a correlation existed between adiponectin levels, gene polymorphisms and long-term oxidative stress in early atherosclerosis T2DM patients of Korean and Han nationalities in the Yanbian region, providing a theoretical genomic basis for effectively preventing various ethnicity-associated macrovascular complications through early clinical antioxidant treatment.

## Subjects and methods

### Subjects and groups

According to the ‘informed consent’ principle, all respondents were recruited from the Jilin Yanbian area and had no genetic relationship with other participants. The individuals were grouped according to the World Health Organization’s criteria for diabetes diagnosis and classification in 1999 (17). Firstly, the normal control group (NGT) consisted of 171 cases with a mean age of 50.93±9.77 years. Secondly, the T2DM group comprised 225 patients with a mean age of 55.89±12.53 years. The T2DM Han nationality carotid atherosclerosis group consisted of 66 cases with an average age of 59.19±10.59 years, while there were 65 Han patients without carotid atherosclerosis that had an average age of 47.12±15.36 years. The Korean nationality carotid atherosclerosis group consisted of 51 cases with average age of 54.67±11.9 years, while there were 43 Korean patients without carotid atherosclerosis that had an average age of 59.30±9.58 years. Finally, the T2DM Korean and Han nationality carotid atherosclerosis positive and negative groups were then divided into two groups according to their genotype (GG/GT or TT). The study was conducted in accordance with the Declaration of Helsinki and with approval from the Ethics Committee of the Affiliated Hospital of Yanbian University (Yanji, China). Written informed consent was provided by all the participants.

### Genomic DNA extraction

A Wizard^®^ Genomic DNA Purification kit (Promega Corporation, Madison, WI, USA) was used for DNA extraction.

### SNP genotyping using the TaqMan probe method

Primers of the adiponectin gene SNP45 were designed according to the reported sequences on the GenBank database and were synthesized by Shanghai GeneCore Biotechnologies Co., Ltd. (Shanghai, China). The upstream and downstream primer sequences were 5′-GCAGCTCCTAGAAAGTAGACTCTGCTG-3′ and 5′-GCAGGTCTGTGATGAAAGAGGCC-3′, respectively.

### TaqMan probe quantitative PCR for the determination of the somatic cell mutation rate

The total mtDNA primer sequences were 5′-GCCTTCCCCCGTAAATGATAT-3′ (forward) and 5′-GAAGAGGAATTGAACCTCTGACTG-3′ (reverse), while the mutated mtDNA primer sequences were 5′-ATTAAAGTCCTACGTGATC-3′ (forward) and 5′-ATGCGATTACCGGGCC-3′ (reverse). The *Taq*Man probe sequence was 5′-TGCCATCTTAACAAACCCTGTTCTTGGGTT-3′ (TaqMan MGB probe and primers were synthesized by GeneCore). A fluorescence sequencing method was used to perform *Taq*Man probe quantitative PCR (completed by Shanghai GeneCore Biotechnologies Co., Ltd.).

### Determination of biochemical indices and IMT

Carbohydrates, triglycerides, high- and low-density lipoproteins and cholesterol, blood glucose and other indicators were determined using an Hitachi 7600-101 automatic biochemical analyzer (Hitachi, Ltd., Tokyo, Japan). An Acuson-128 probe (Providian Medical Equipment LLC, Willowick, OH, USA) with a frequency of 7.5 MHz Doppler was used to measure the carotid IMT at six different places. The points selected were as follows: Two points on each side of the walls of the carotid artery at the level of the carotid artery enlargement, 1.0 cm above and 1.0 cm underneath the carotid artery enlargement. The average of these six values was used as the value of the carotid artery IMT. IMT values of ≥1.0 mm, and/or hard or soft spots were considered to be a positive diagnosis.

### Statistical analysis

A gene counting method was used to calculate the frequencies of genotypes and alleles in each group and the Hardy-Weinberg equilibrium was used to analyze the representation of the samples. Risk factors of carotid atherosclerosis lesions were screened by logistic regression analysis, while genotype and allele frequencies in these groups were analyzed using the χ^2^ test. One-way analysis of variance was used to analyze multiple sets of measurement data. Results are expressed as the mean ± SD and P<0.05 was considered to indicate a statistically significant difference. Statistical analyses were performed using the SPSS 17.0 statistical software (SPSS, Inc. Chicago, IL, USA).

## Results

### Adiponectin SNP45 genotyping results

**Figure 1:**
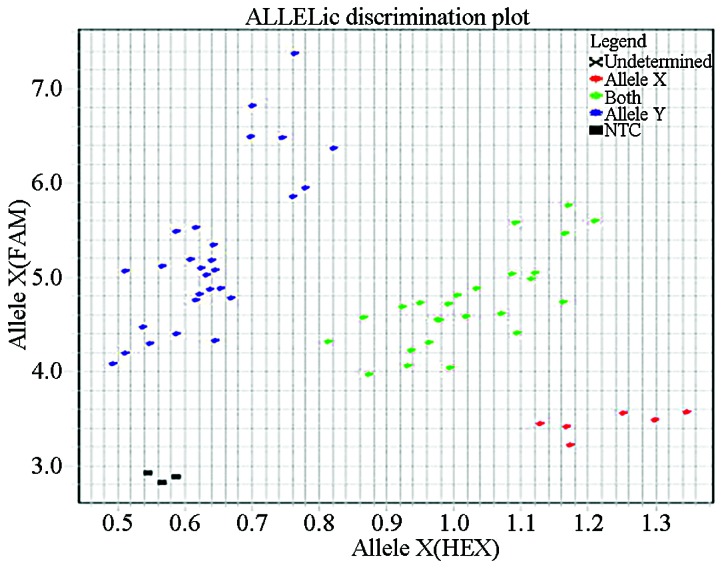
SNP genotyping results determined using the *Taq*Man probe method. Abscissa, HEX signal strength; ordinate, FAM signal strength; NTC, no template control; black spots, control; undetermined, unrecognized; red points, GG homozygotes; green points, TG heterozygotes; blue points, TT homozygotes; SNP, single nucleotide polymorphism. Hex in the x-axis shows the signals marked with a hex probe which are detected. The further from the origin, the stronger the signal; Fax in the y-axis shows the signals that are marked with a fam probe which are detected. The farther from the origin, the stronger the signal.

Results from SNP genotyping using the *Taq*Man probe method indicated that the SNP45 was divided into GG, TG and TT genotypes. As shown in Fig. 1, red points indicate SNP45 GG homozygotes, green points represent TG heterozygotes and blue points indicate TT homozygotes.

### Adiponectin SNP45 sequencing diagram

**Figure 2:**
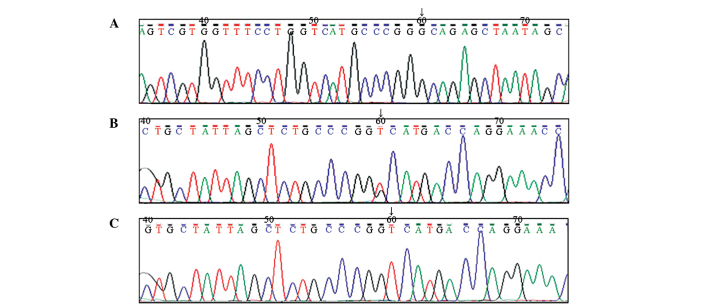
Sequencing figures demonstrating (A) homozygous GG, (B) heterozygous TG and (C) homozygous TT genotypes.

As shown in Fig. 2, peaks demonstrated SNP homozygous GG (Fig 2A), heterozygous TG (Fig. 2B) and homozygous TT (Fig. 2C) genotypes.

### Somatic cell mutation rate of acquired mtDNA A3243G

**Figure 3:**
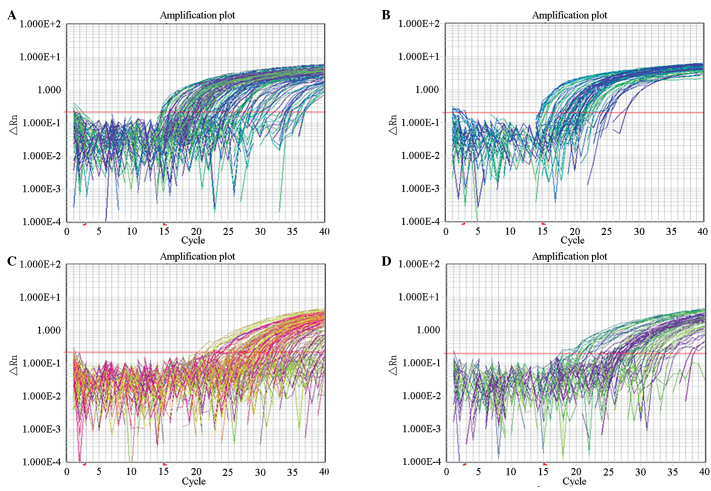
(A) Total and (B) mutated mtDNA gene data amplified fluorescence curves. A standard curve was calculated using the standards of the specified known copy number. Slope values were used to evaluate the amplification efficiency of PCR; when the amplification efficiency was 100%, the slope was −3.33, for 10-fold dilution of the standards, the gradient of the Ct value was 3.33 Cts. The intercept of the standard curve on the y-axis was the Ct value. The R^2^ value was used to describe the correlation between the Ct value of the standards forming the standard calibration curve and the logarithmic value of the initial template copy number. The value was between 0 and 1 and the closer the value was to 1, the better the correlation between the Ct values and their starting template copy number. Ct value, the number of PCR cycles experienced by each reaction tube fluorescent signals had reached the set threshold; PCR, polymerase chain reaction; mtDNA, mitochondrial DNA.

Fig. 3A shows the total mtDNA gene data amplified fluorescence curve, while Fig. 3B shows the mutated mtDNA gene data amplified fluorescence curve. The somatic cell mutation rate of acquired mtDNA A3243G was calculated according to the following example shown for the normal control group 1. Mutation rate=1/2^ctm-ctt^, where, ct_m_=(ct_m1_+ct_m2_+ct_m3_)/3 and ct_t_=(ct_t1_+ct_t2_+ct_t3_)/3. ct_m1_+ct_m2_+ct_m3_ in the normal contro1 group 1 all come from mututal mtDNA Gene Data and ct_t1_, ct_t2_, ct_t3_ in the normal control group 1 and the dates all come from the total mtDNA Gene Data.

### Comparison of acquired mtDNA A3243A/G mutation rates between the normal and T2DM groups

Statistically significant differences were observed in the mutation rates of acquired mtDNA A3243A/G between the normal and T2DM patients from the Yanbian region (P<0.05; Table I).

### Comparison of acquired mtDNA A3243A/G mutation rates between T2DM patients with or without carotid atherosclerosis

Statistically significant differences were observed when comparing the acquired mtDNA A3243A/G mutation rates between T2DM patients with and without carotid atherosclerosis in the Yanbian area (P<0.05; Table II).

### Comparison of acquired mtDNA A3243A/G somatic cell mutation rates in T2DM patients with adiponectin SNP45 TT or TG/GG genotypes

Statistically significant differences were observed when comparing the acquired mtDNA A3243A/G somatic cell mutation rates in T2DM patients with adiponectin gene SNP45 TT or TG/GG genotypes in the Yanbian area (P<0.05; Table III).

### Comparison of the acquired mtDNA A3243A/G somatic cell mutation rates in T2DM patients with carotid atherosclerosis and adiponectin SNP45 T/G genotypes

Statistically significant differences were observed when comparing the mtDNA A3243A/G somatic mutation rates of T2DM carotid atherosclerosis patients with adiponectin SNP45 TT and TG/GG genotypes (P<0.05; Table IV).

### Comparison of adiponectin SNP45 T/G genotypes and acquired mtDNA A3243A/G somatic cell mutation rates in T2DM patients with carotid atherosclerosis of Han and Korean nationalities in the Yanbian area

Statistically significant differences were not observed when comparing adiponectin SNP45 TT or TG/GG genotypes and the mtDNA A3243A/G somatic mutation rates of T2DM carotid atherosclerosis patients of Han and Korean nationalities in the Yanbian area (P>0.05; Table V).

## Discussion

Diabetic patients have high blood sugar levels, thus, the body is exposed to long-term oxidative stress. mtDNA oxidative stress induces cellular damage. Mitochondria are the main site of cellular biological oxidation and energy conversion, and are not only a major source of reactive oxygen species (ROS), but are also the main target for ROS damage (18). Acquired mtDNA A3243G somatic cell mutation rates, determined by the *Taq*Man probe quantitative PCR method, reflect the long-term level of oxidative stress in patients with T2DM (1). The results of the present study revealed that significant differences existed in the mtDNA A3243A/G somatic cell mutation rates between the NGT and T2DM groups (P<0.05), indicating that T2DM in the Yanbian area is associated with long-term oxidative stress. Oxidative stress is the main regulator of various signal transduction systems involved in atherosclerotic vascular inflammation and throughout the whole formation process of fatty streaks and lesions progressing to final plaque rupture. Comparisons between the acquired mtDNA A3243A/G somatic cell mutation rates in T2DM patients with and without carotid atherosclerosis in the Yanbian area exhibited statistically significant differences (P<0.05), indicating that T2DM with carotid atherosclerosis is associated with long-term oxidative stress.

Adiponectin may regulate the development of T2DM and chronic complications, including macrovascular diseases, through oxidative stress (6). Previous studies have demonstrated that adiponectin is associated with short-term oxidative stress, however, to the best of our knowledge, there have been no studies investigating the association between adiponectin gene polymorphisms, long-term oxidative stress and macrovascular lesions in T2DM patients (1,3–7). The results of the current study revealed that the acquired mtDNA A3243A/G somatic cell mutation rates of T2DM patients with adiponectin SNP45 TT and TG/GG genotypes in the Yanbian region exhibited statistically significant differences (P<0.05). This observation indicates that the adiponectin SNP45 T/G genotype is associated with long-term oxidative stress in patients with T2DM. In addition, the results revealed that there were no statistically significant differences in the acquired mtDNA A3243A/G somatic cell mutation rates of T2DM carotid atherosclerosis patients with SNP45 TT and TG/GG genotypes of Korean and Han nationalities in the Yanbian region (P>0.05). Therefore, the results indicate that the adiponectin SNP45 T/G genotype and long-term oxidative stress may be associated with carotid atherosclerosis in T2DM patients of Korean and Han nationalities in the Yanbian region. The G allele was a risk factor, but no ethnic differences were identified between the Korean and Han populations.

In conclusion, the mtDNA A3243A/G somatic cell mutation rates of T2DM patients with carotid atherosclerosis were significantly higher compared with patients without carotid atherosclerosis, indicating that long-term oxidative stress may be involved in carotid atherosclerosis of T2DM. In addition, the adiponectin SNP45 T/G genotype correlated with the mtDNA A3243A/G somatic mutation rates of patients with T2DM and carotid atherosclerosis in the Yanbian region, indicating that the G allele was a risk factor for T2DM. However, no ethnic differences were identified between the Korean and Han populations. Therefore, the results of the present study indicate that adiponectin gene polymorphisms may lead to diabetes atherosclerosis through oxidative stress.

## Figures and Tables

**Table I:** Comparison of the acquired mtDNA A3243A/G somatic cell mutation rates in the NGT and T2DM groups.

Groups	Cases, n	mtDNA 3243 somatic cell mutation rate, %
NGT	171	0.046±0.045
T2DM	255	0.739±0.738

NGT, normal control; T2DM, type II diabetes mellitus; mtDNA, mitochondrial DNA.

**Table II:** Comparison of the acquired mtDNA A3243A/G somatic cell mutation rates in T2DM patients with and without carotid atherosclerosis.

T2DM groups	Cases, n	mtDNA 3243 somatic cell mutation rate, %
Carotid atherosclerosis negative	108	0.279±0.255
Carotid atherosclerosis positive	117	0.938±0.565

T2DM, type II diabetes mellitus; mtDNA, mitochondrial DNA.

**Table III:** Comparison of the acquired mtDNA A3243A/G somatic mutation rates in T2DM patients with adiponectin SNP45 TT and TG/GG genotypes.

T2DM groups	Cases, n	mtDNA 3243 somatic cell mutation rate, %
TT	100	0.596±0.650
TG/GG	125	1.061±0.532

T2DM, type II diabetes mellitus; mtDNA, mitochondrial DNA; SNP, single nucleotide polymorphism.

**Table IV:** Comparison of the acquired mtDNA A3243A/G somatic mutation rates in T2DM carotid atherosclerosis patients with adiponectin SNP45 T/G genotypes.

T2DM carotid atherosclerosis group	Cases, n	mtDNA 3243 somatic cell mutation rate, %
TT	60	0.279±0.255
TG/GG	57	0.938±0.565

mtDNA, mitochondrial DNA; T2DM, type II diabetes mellitus; SNP, single nucleotide polymorphism.

**Table V:** Comparison of adiponectin SNP45 T/G genotypes and acquired mtDNA A3243A/G somatic mutation rates in T2DM patients with carotid atherosclerosis of Han and Korean nationalities in the Yanbian region.

Carotid atherosclerosis group	TT cases, n	Variability	TG/GG cases, n	Mutation rate
Han nationality	30	0.209±0.150	36	0.332±0.455
Korean nationality	30	0.390±0.467	21	0.335±0.491

SNP, single nucleotide polymorphism; mtDNA, mitochondrial DNA; T2DM, type II diabetes mellitus.

## References

[1] Nomiyama T, Tanaka Y, Hattori N (2002). Accumulation of somatic mutation in mitochondrial DNA extracted from peripheral blood cells in diabetic patients. Diabetologia.

[2] Nishikawa T, Edelstein D, Du XL (2000). Normalizing mitochondrial superoxide production blocks three pathways of hyperglycaemic damage. Nature.

[3] Furukawa K, Hori M, Ouchi N (2004). Adiponectin down-regulates acyl-coenzyme A: cholesterol acyltransferase-1 in cultured human monocyte-derived macrophages. Biochem Biophys Res Commun.

[4] Hattori S, Hattori Y, Kasai K (2005). Hypoadiponectinemia is caused by chronic blockade of nitric oxide synthesis in rats. Metabolism.

[5] Hattori Y, Akimoto K, Gross SS, Hattori S, Kasai K (2005). Angiotensin-II-induced oxidative stress elicits hypoadiponectinaemia in rats. Diabetologia.

[6] Motoshima H, Wu X, Mahadev K, Goldstein BJ (2004). Adiponectin suppresses proliferation and superoxide generation and enhances eNOS activity in endothelial cells treated with oxidized LDL. Biochem Biophys Res Commun.

[7] Steffes MW, Gross MD, Schreiner PJ (2004). Serum adiponectin in young adults - interactions with central adiposity, circulating levels of glucose, and insulin resistance: the CARDIA study. Ann Epidemiol.

[8] Ouchi N, Kihara S, Arita Y (2000). Adiponectin an adipocyte-derived plasma protein, inhibits endothelial NF-kappaB signaling through a cAMP-dependent pathway. Circulation.

[9] Matsuda M, Shimomura I, Sata M (2002). Role of adiponectin in preventing vascular stenosis: the missing link of adipo-vascular axis. J Biol Chem.

[10] Fernandez M, Triplitt C, Wajcberg E (2008). Addition of pioglitazone and ramipril to intensive insulin therapy in type 2 diabetic patients improves vascular dysfunction by different mechanisms. Diabetes Care.

[11] Lacquemant C, Froguel P, Lobbens S, Izzo P, Dina C, Ruiz J (2004). The adiponectin gene SNP+45 is associated with coronary artery disease in type 2 (non-insulin-dependent) diabetes mellitus. Diabet Med.

[12] Qi L, Li T, Rimm E (2005). The +276 polymorphism of the APM-1 gene, plasma adiponectin concentration, and cardiovascular risk in diabetic men. Diabetes.

[13] Qi L, Doria A, Manson JE (2006). Adiponectin genetic variability, plasma adiponectin, and cardiovascular risk in patients with type 2 diabetes. Diabetes.

[14] Bacci S, Menzaghi C, Ercolino T (2004). The +276 G/T single nucleotide polymorphism of the adiponectin gene is associated with coronary artery disease in type 2 diabetic patients. Diabetes Care.

[15] Filippi E, Sentinelli F, Romeo S (2005). The adiponectin gene SNP+276G>T associates with early-onset coronary artery disease and with lower levels of adiponectin in younger coronary artery disease patients (age <or=50 years). J Mol Med (Berl).

[16] Ma X, Lin Y, Li J (2008). Adiponectin gene polymorphisms are associated with the risk of coronary artery disease in Chinese type 2 diabetic patients. Zhong Guo Tang Niao Bing Za Zhi She.

[17] World Health Organization, Geneva (1999). Definition, Diagnosis and Classification of Diabetes Mellitus and its Complications.

[18] Li N, Frigerio F, Maechler P (2008). The sensitivity of pancreatic beta-cells to mitochondrial injuries triggered by lipotoxicity and oxidative stress. Biochem Soc Trans.

